# Uniform Water Potential Induced by Salt, Alkali, and Drought Stresses Has Different Impacts on the Seedling of *Hordeum jubatum*: From Growth, Photosynthesis, and Chlorophyll Fluorescence

**DOI:** 10.3389/fpls.2021.733236

**Published:** 2021-09-30

**Authors:** Congcong Shi, Fan Yang, Zihao Liu, Yueming Li, Xiaolin Di, Jinghong Wang, Jixiang Lin

**Affiliations:** ^1^College of Landscape Architecture, Northeast Forestry University, Harbin, China; ^2^Key Laboratory of Saline-Alkali Vegetation Ecology Restoration, Ministry of Education, Northeast Forestry University, Harbin, China

**Keywords:** *Hordeum jubatum*, salt, alkali, drought, water potential, photosynthesis, chlorophyll fluorescence

## Abstract

Hordeum jubatum is a halophyte ornamental plant wildly distributed in the Northeast of China, where the low water potential induced by various abiotic stresses is a major factor limiting plant growth and development. However, little is known about the comparative effects of salt, alkali, and drought stresses at uniform water potential on the plants. In the present study, the growth, gas exchange parameters, photosynthetic pigments, and chlorophyll fluorescence in the seedlings of *H. jubatum* under three low water potentials were measured. The results showed that the growth and photosynthetic parameters under these stresses were all decreased except for carotenoid (Car) with the increasing of stress concentration, and alkali stress caused the most damaging effects on the seedlings. The decreased net photosynthetic rate (*P*n), stomatal conductance (*G*s), and intercellular CO_2_ concentrations (*C*i) values under salt stress were mainly attributed to stomatal factors, while non-stomatal factors were dominate under drought and alkali stresses. The reduced chlorophyll and slightly increased Car contents occurred under these stresses, and most significant changed under alkali stress. In addition, the maximum photochemical efficiency (Fv/Fm), actual photochemical efficiency (ΦPSII), and photochemical quenching coefficient (q_P_) under the stresses were all decreased, indicating that salt, alkali, and drought stresses all increased susceptibility of PSII to photoinhibition, reduced the photosynthetic activity by the declined absorption of light for photochemistry, and increased PSII active reaction centers. Moreover, the non-photochemical quenching coefficient (NPQ) of alkali stress was different from salt and drought stresses, showing that the high pH of alkali stress caused more damaging effects on the photoprotection mechanism depending on the xanthophyll cycle. The above results suggest that the *H. jubatum* has stronger tolerance of salt than drought and alkali stresses, and the negative effects of alkali stress on the growth and photosynthetic performance of this species was most serious.

## Introduction

Soil salinization, alkalization, and drought have been considered as serious environmental hazards to the agricultural, grassland, and urban ecosystems, which also severely affects plant growth and productivity worldwide (Sdouga et al., 2019; Barnard et al., 2021). It is estimated that more than 10% of total global land has suffered from salinization, and the area is still expanding (Farooq et al., 2015). In addition, soil salinization is often accompanied by the occurrence of alkalization in the northeast of China, where alkaline salts (e.g., NaHCO_3_) are the main components in the soil (Yin et al., 2019). As another important abiotic stress, it has been reported that drought would affect 50% of the global arable land, and drought stress is often caused indirectly by soil salinization and alkalization, which damage the growth and metabolism process of plants (Igiehon and Babalola, 2021).

*Hordeum jubatum* is a biennial herb of the Gramineae family. It is native to the cold temperate zone of North America and Eurasia, and is widely distributed in northeastern China. This plant is a salt-tolerant species, which can survive in the soil with high concentration salts (Israelsen et al., 2011). In addition, *H. jubatum* also has good drought tolerance, its seed can germinate under very low water potential conditions (Zou et al., 2018). Due to the good stress resistance under these adverse environments, this plant plays an important role in soil improvement and ecological restoration in the northeast of China. However, no information is available on the stress tolerance mechanism under saline–alkali and drought stresses during the early seedling stage.

The early seedling stage is an extremely crucial period of plants, and is susceptible to the adverse environments (Wang et al., 2019; Ocvirk et al., 2020; Ren et al., 2020). During this period, water absorption is a determining factor and it regulates various physiological processes in the plant (Klugl and Di Pietro, 2021). In general, both salt-alkali and drought stresses can induce lower water potential, which limits water uptake and inhibits growth of plants. Salt stress disturbs the ion balance and nutrient uptake due to osmotic stress and toxic ions (accumulation of excessive Na^+^), ultimately affecting normal physiological processes (Xu et al., 2018). Previous studies have demonstrated that alkali stress and salt stress are significantly different. Alkali stress has not only the same damaging factors as salt stress but also adds high-pH stress, leading to more destruction to the plant (Lin et al., 2016; Song et al., 2017). Additionally, drought stress directly causes soil water deficit, which leads to a decreased ability of leaf water absorption and excessive transpiration, thereby inhibiting plant growth (Chmielewska et al., 2016). Most studies have mainly comparatively analyzed the effects of salt and drought stresses (Elbasan et al., 2020; He et al., 2021) or salt and alkali stresses (Hu et al., 2019; Jia et al., 2019) on the plant, but little is known on the effects of salt, alkali, and drought stresses simultaneously on plants based on the uniform water potential.

Photosynthesis is one of the most important physiological processes in the carbon assimilation of plants. Photosynthetic capacity can affect the primary productivity and measure the growth level of plants under stress conditions. Generally, salt stress decreases the activity of the PSII reaction center of the leaf. Massive accumulation of surplus electrons in the electron transport chain due to a decrease of electron transfer rates causes electron leakage and then accelerates the degree of breakage of PSII reaction center under salt stress (Zhang et al., 2017; Hou et al., 2018). In addition, Wang et al. (2020) have clarified that compared with salt stress, alkali stress decreased the photosynthetic electron transport rate and stomatal conductance, and also reduced the aerenchyma amount, and then influenced the CO_2_ influx into mesophyll cells of *Leymus chinensis*. Drought stress directly makes the stomata closed, resulting in a reduction of CO_2_ absorption and transportation of non-structural carbon (NSC), which is an essential element of the photosynthetic system (Steppe et al., 2015; Hajiboland et al., 2017). The above results suggest that photosynthesis responses of plant to salt, alkali, and drought stresses are markedly different. Therefore, it is of great scientific significance to explore the physiological mechanism of plant photosynthetic systems under such conditions.

Therefore, in the present study, we comparatively investigated seedling growth, photosynthesis, photosynthetic pigments, and chlorophyll fluorescence of *H. jubatum* under salt, alkali, and drought stresses based on the same water potential. The aims were to explore the response of growth and photosynthetic performance to a wide range of low water potentials caused by the three stresses and clarify their different stress features.

## Materials and Methods

### Plant Materials and Growth Condition

The mature seeds of *H. jubatum* used in this pot-controlled experiment were provided by Beijing Zhengdao Seed Industry Company. The same size, full, and healthy seeds were selected as experiment materials. The seeds were soaked and disinfected with 1% NaClO for 20 min, then rinsed with distilled water three times and dried in the shade for future use. Thirty seeds were evenly sown in the plastic pot (17 cm in height, 8 cm in diameter) containing 2.5 kg farmland soil. The farmland physical and chemical soil properties were: 30.62% of soil water content, 16.61 g kg^–1^ of soil organic matter, 0.64 g kg^–1^ of total nitrogen, 0.05 g kg^–1^ of available nitrogen, 0.04 g kg^–1^ of available phosphorus, 0.11 g kg^–1^ of available potassium, and the pH is 7.02. The pots were cultivated in a greenhouse for germination, where the average temperature and light time were 25°C and 12 h, respectively.

### Experimental Design

Three abiotic stress factors including salt, alkali, and drought were set up in this experiment. Three salt concentrations 50, 100, and 200 mM (S1, S2, and S3) were applied, and the corresponding water potentials were −0.21, −0.47, and −0.82 MPa, respectively. To maintain uniform water potential, three alkali (NaHCO_3_) concentrations were also determined by Water Potential Meter (WESCOR PSYPRO, United States), which were marked as A1, A2, and A3, respectively. In addition, three concentrations of PEG-6000 solution (D1, D2, and D3) were also configured to simulate drought stress based on the above water potentials (Michel and Kaufmann, 1973). Three replicates were set for each treatment, and there was a total of 30 pots of plants in the present research. When the seedlings were 40 days old, each pot was irrigated with 150 mL of different stress solutions. After stress treatment for 5 days, the growth parameters, photosynthetic parameters, and chlorophyll fluorescence parameters were measured.

### Growth Measurement

The fresh weight (FW) of shoot per pot was immediately weighed when seedlings of *H. jubatum* were harvested. The shoots were placed in an oven at 105°C for 10 min, and then dried at 65°C until a constant dry weight (DW) was obtained. The water content (WC) of each treatment was calculated as the formula: (FW - DW)/FW × 100%.

### Gas Exchange Parameters

After 46 days of seedling growth of *H. jubatum*, four fully expanded leaves were randomly selected to measure gas exchange parameters per pot. The open flow LI-6400XT gas-exchange system (LI-COR Biosciences, Lincoln, NE, United States) was used to determine the net photosynthetic rate (*P*n), stomatal conductance (*G*s), intercellular CO_2_ concentration (*C*i), transpiration rate (*T*r), and water use efficiency (*WUE*) between 9:00 and 11:30 am. The CO_2_ concentration, average temperature, flow rate and light flux intensity in the environment, and block temperature were 420 μmol mol^–1^, 25°C, 500 μmol s^–1^, 64 μmol m^–2^ s^–1^, and 28.5°C, respectively.

### Chlorophyll Fluorescence

After measuring various gas exchange parameters, nine leaves were randomly selected from each treatment to determinate chlorophyll fluorescence indicators using FluorCam FC 800-O fluorescence imaging system (Photon System Instruments, Czechia). Before measurement, all samples need to be dark-adapted for at least 30 min to ensure that the PSII reaction center is completely open. After dark adaptation, the samples were placed in FluorCam CCD camera, where four LED panels provided measuring flashes, actinic lights, and saturating pulses.

The FluorCAM program was used to obtain a series of required fluorescence parameters by analyzing the fluorescence image of each treatment. The maximum photochemical efficiency (Fv/Fm), actual photochemical efficiency (ΦPSII), photochemical quenching coefficient (q_P_), and non-photochemical quenching coefficient (NPQ) were calculated according to Lazar (2003).

### Photosynthetic Pigments

From each treatment, 0.05 g of fresh leaves was cut into small pieces and put in a centrifuge tube including 10 mL of 80% acetone. All the centrifuge tubes were placed in the dark for 48 h until the leaf photosynthetic pigment was completely extracted. The absorbance of supernatant was determined using an ultraviolet-visible spectrophotometer (BioMate 3S UV-visible, Thermo Fisher Scientific, United States) at wavelengths of 663, 645, and 440 nm, respectively. The contents of chlorophyll (Chl a and Chl b) and carotenoid (Car) were calculated as the formulas described in Gitelson et al. (2003).

### Statistical Analysis

The data used for comparison between each treatment are all the average values of each treatment, and SPSS 26.0 (SPSS Inc., Chicago, IL, United States) software was used for data comparison and analysis between each treatment. One-way analysis of variance (ANOVA) was used to analyze the average value and the comparative relationship between the treatments. Two-way ANOVA was used to analyze the effects of water potential, stress type and their interactions on the biomass and photosynthetic indexes of *H. jubatum* seedlings, and the significant difference between treatments was analyzed by Duncan’s multiple range test (*P* < 0.05). Sigmaplot 13.0 software was used to plot the data between each treatment.

## Results

### Plant Growth

**FIGURE 1:**
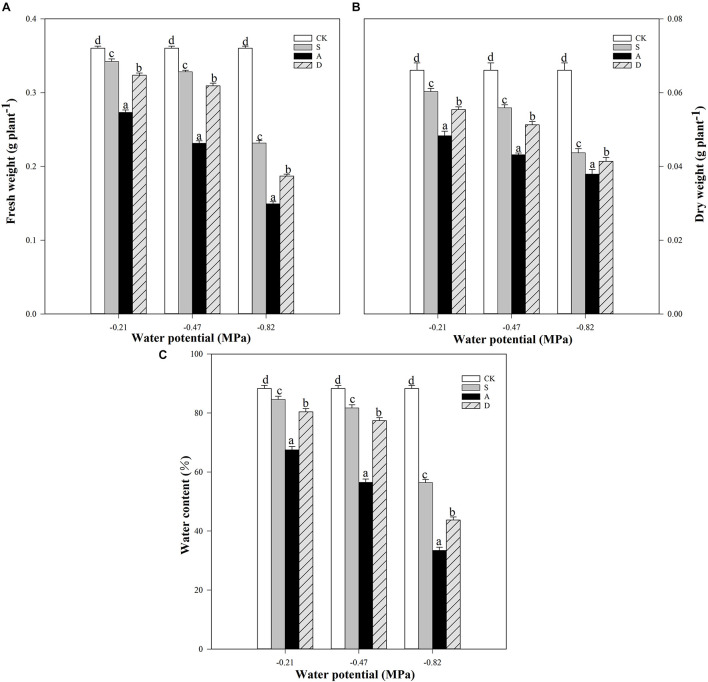
Fresh weight (FW, **A**), dry weight (DW, **B**), and water content (WC, **C**) of *Hordeum jubatum* under different water potentials induced by salt, alkali, and drought stresses. Different small letters indicate significant difference of salt, alkali, and drought stresses under uniform water potential. The differences in each parameter were detected by one-way ANOVA at *P* < 0.05 level. Bars represent mean ± SE (*n* = 3).

Two-way ANOVA results showed that the FW, DW, and WC were significantly affected by water potential, stress type, and their interaction (*P* < 0.01; Table 1). With the increasing stress concentration, both FWs and DWs were significantly decreased at different water potentials, and the reductions under −0.82 MPa were higher than those under other water potentials. For example, the FW and DW of salt stress at −0.82 MPa decreased by 36 and 34%, respectively, compared to the control groups, but only decreased 8.9, 5%, and 15, 8.7%, respectively, under −0.47 and −0.21 MPa (Figures 1A,B). Moreover, the FWs and DWs of alkali stress were the lowest compared to other treatments under all the water potentials. The WCs of different stresses also showed a similar trend with FWs and DWs under the three water potentials (Figure 1C).

**TABLE 1:** Two-way ANOVA of the effects of water potential (WP), stress type (ST), and their interactions on the growth, photosynthesis, photosynthetic pigments, and chlorophyll fluorescence indexes of *Hordeum jubatum* seedlings.

Dependent variable	Independent variable		
	*WP*	*ST*	*WP* × *ST*
FW (g plant^–1^)	1179.321644***	294.295445***	24.417131***
DW (g plant^–1^)	195.109***	47.530***	5.452**
WC (%)	800.612***	198.364***	18.611***
*P*n (μmol CO_2_ m^–2^ s^–1^)	396.681***	28.942***	5.487**
*G*s (mol H_2_O m^–2^ s^–1^)	15.578***	563.445***	7.982***
*C*i (μmol mol^–1^)	57.771***	121.158***	10.433***
*T*r (mmol H_2_O m^–2^ s^–1^)	156.763***	33.690***	4.639**
*WUE* (μmol CO_2_ mmolH_2_O^–1^)	14.705***	0.248^NS^	0.451^NS^
Chl a (mg g^–1^)	45.979***	7.167123**	3.571*
Chl b (mg g^–1^)	35.992***	0.178**	0.374^NS^
Car (mg g^–1^)	4.531*	5.973*	0.482^NS^
Fv/Fm (ratio)	12.988***	8.402**	0.327^NS^
ΦPSII (ratio)	42.902***	10.786***	1.460^NS^
NPQ (ratio)	34.780***	21.885***	4.142**
q_P_ (ratio)	44.448***	5.143***	1.133**

Date represent *F*-values at 0.05 level. *, **, ***, and NS indicate significance at *P* < 0.05, *P* < 0.01, *P* < 0.001, and *P* > 0.05, respectively.

### Gas Exchange Parameters

Two-way ANOVA results showed that the net photosynthetic rates (*P*n) and transpiration rate (*T*r) were affected by water potential (*P* < 0.001), stress type (*P* < 0.001), and their interaction (*P* < 0.01). The stomatal conductance (*G*s) and intercellular CO_2_ concentration (*C*i) were significantly affected by water potential, stress type, and their interaction (*P* < 0.001). However, the *WUE* was only significantly affected by water potential (*P* < 0.001; Table 1).

**FIGURE 2:**
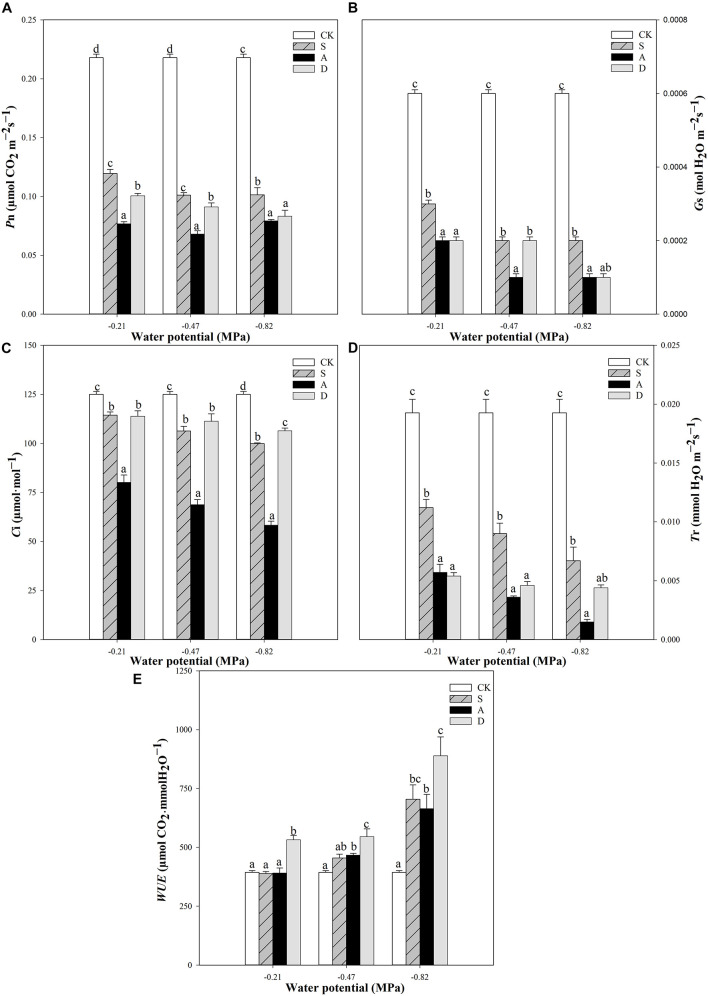
Photosynthetic rate (*P*n, **A**), stomatal conductance (*G*s, **B**), intercellular CO_2_ concentration (*C*i, **C**), transpiration rate (*T* r, **D**), and the water use efficiency (WUE, **E**) of *Hordeum jubatum* under different water potentials induced by salt, alkali, and drought stresses. Different small letters indicate significant difference of salt, alkali, and drought stresses under uniform water potential. The differences in each parameter were detected by one-way ANOVA at *P* < 0.05 level. Bars represent mean ± SE (*n* = 3).

With the increase of stress concentration, the values of *P*n, *G*s, *C*i, and *T*r all decreased in the three types of stresses except for the *P*n at alkali stress, which had a slight increase from the −0.47 to −0.82 MPa (Figures 2A–D). The trend was more obvious under alkali stress. Adversely, with the increasing stress concentration, the value of *WUE* increased in the stresses, and more markedly under drought stress (Figure 2E).

### Chlorophyll Fluorescence

Two-way ANOVA results showed that the ΦPSII, NPQ, and q_P_ were significantly affected by water potential and stress type (*P* < 0.001). In addition, the Fv/Fm, ΦPSII were not affected by the interaction of water potential and stress type (*P* > 0.05), but the NPQ and q_P_ were affected by the interaction of water potential and stress type (*P* < 0.01; Table 1).

**FIGURE 3:**
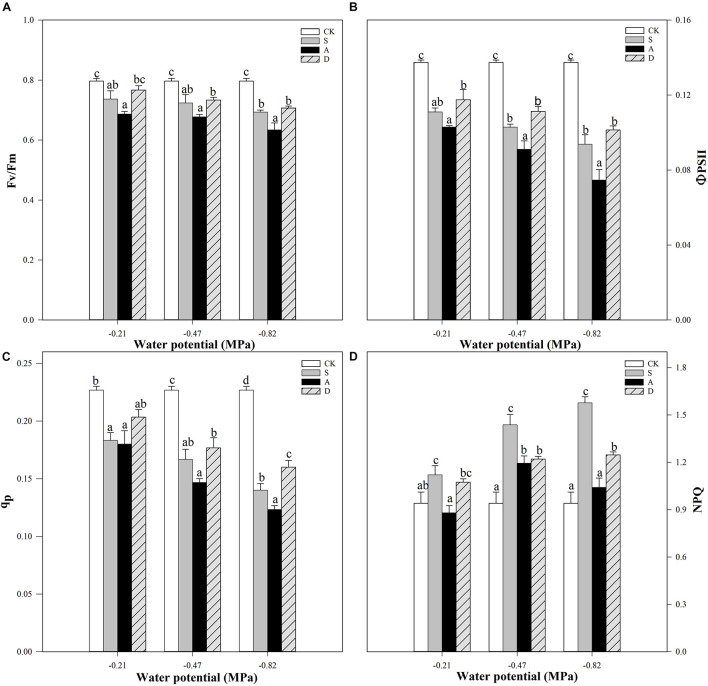
Apparent quantum yield of PSII (Fv/Fm, **A**), actual quantum yield of photochemical energy conversion in PSII (ΦPSII, **B**), photochemical quenching coefficient (qP, **C**), and non-photochemical quenching coefficient (NPQ, **D**) of *Hordeum jubatum* under different water potentials induced by salt, alkali, and drought stresses. Different small letters indicate significant difference of salt, alkali, and drought stresses under uniform water potential. The differences in each parameter were detected by one-way ANOVA at *P* < 0.05 level. Bars represent mean ± SE (*n* = 3).

With the increase of stress concentration, the Fv/Fm, ΦPSII, and q_P_ showed an obvious decreasing trend under the stresses, while the NPQ increased first and then decreased (Figure 3). For Fv/Fm and ΦPSII, the decline was more markedly under alkali stress with the decreasing water potential (*P* < 0.05). For example, the Fv/Fm of S3, A3, and D3 treatments decreased 13.0, 20.5, and 11.3%, respectively, compared to the control group (CK) at −0.82 MPa, and the ΦPSII of S3, A3, and D3 treatments decreased 31.7, 45.7, and 26.2%, respectively, under this water potential (Figures 3A,B). Moreover, the q_P_ of S1 and A1 treatments decreased 38.2 and 45.6%, respectively, in comparison to the CK, which had no significant difference (*P* > 0.05), but the q_P_ of A2 and A3 treatments was more significantly declined than that of S2 and S3 treatments (*P* > 0.05; Figure 3C). In addition, the NPQ of salt and drought stresses both increased and that of salt stress was higher than drought stress with the decreasing of water potential (Figure 3D). Differently, the NPQ values of the alkali stress under different water potentials were all evidently lower than that of salt and drought stresses and reached the highest at −0.47 MPa.

### Photosynthetic Pigments

Two-way ANOVA analysis showed that the chlorophyll a (Chl a) content and chlorophyll b (Chl b) content were affected by water potential (*P* < 0.001) and stress type (*P* < 0.01), while the Chl a was affected by the interaction of water potential and stress type (*P* < 0.05) and Chl b was not affected by their interaction (*P* > 0.05). Unlike Chl a and Chl b, the Car content was affected by water potential (*P* < 0.05) and stress type (*P* < 0.05), but not affected by the interaction of water potential and stress type (*P* > 0.05; Table 1).

**FIGURE 4:**
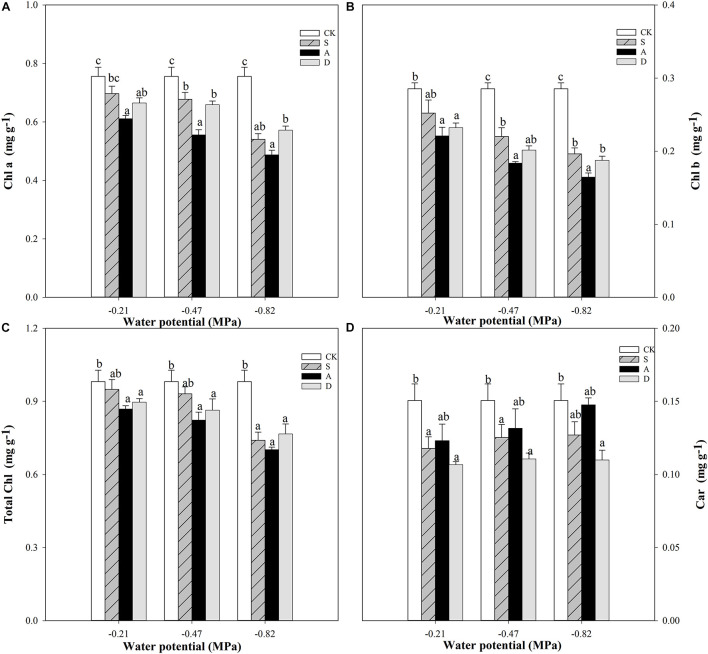
Chlorophyll a (Chl a, **A**), chlorophyll b (Chl b, **B**), total chlorophyll (Total Chl, **C**), and carotenoid (Car, **D**) of *Hordeum jubatum* under different water potentials induced by salt, alkali, and drought stresses. Different small letters indicate significant difference of salt, alkali, and drought stresses under uniform water potential. The differences in each parameter were detected by one-way ANOVA at *P* < 0.05 level. Bars represent mean ± SE (*n* = 3).

With the increase of stress concentration, the Chl a, Chl b, and total chlorophyll (total Chl) contents were all decreased under the three stresses, but the Car was slightly increased. The effect was the most obvious under the alkali stress (Figure 4). The most significant declines of Chl a and Chl b were alkali stress groups compared to the CK under different water potentials (Figures 4A,B). In addition, the largest reduction of total Chl was alkali treatments in comparison to the CK, and which salt treatments were higher than drought treatments except for S3 and D3 under different water potentials (Figure 4C). Unlike chlorophyll content, the Car content of different stresses showed an increasing trend with the decrease of water potential, but the differences among the three stresses were not significant (*P* ≥ 0.05; Figure 4D).

### Correlation Analysis Among Plant Growth, Photosynthetic and Fluorescence Indexes

Correlation analysis results showed that the FW, DW, and WC of salt stress had obvious correlation with the photosynthetic and fluorescence parameters except for Car and q_P_ under different water potentials. In addition, the *WUE* and NPQ of salt stress had negative correlation with the growth parameters. For photosynthetic parameters, the *P*n of salt stress had significantly positive correlation with *G*s, *C*i, *T*r, and Chl b under different water potentials (*P* < 0.01). Moreover, the Fv/Fm of salt stress had significant positive correlation with ΦPSII, and the NPQ of salt stress had significant negative correlation with ΦPSII and Fv/Fm (*P* < 0.01; Table 2). A similar trend was shown in the growth parameters of alkali stress under different water potentials except for the DW, which had positive correlation with q_P_. The *P*n was shown that had significant positive correlation with *G*s, *C*i, *T*r, Chl a, and Chl b (*P* < 0.01). Unlike salt stress, the *WUE* of alkali stress had no correlation with *G*s and *T*r under different water potentials (Table 3). For the growth parameters, it should be noted that the DW of drought stress was positively correlated with Car under different water potentials, which was different from salt and alkali stresses (Table 4).

**TABLE 2:** Correlation analysis between plant growth and photosynthetic parameters of salt stress under different water potentials.

Correlations	FW	DW	WC	*P*n	*G*s	*C*i	*T*r	*WUE*	Chl a	Chl b	Car	Fv/Fm	ΦPSII	NPQ	q_P_
FW	1														
DW	0.957**	1													
WC	0.998**	0.939**	1												
*P*n	0.584*	0.734**	0.548	1											
*G*s	0.718**	0.830**	0.688*	0.969**	1										
*C*i	0.831**	0.876**	0.813**	0.875**	0.935**	1									
*T*r	0.711**	0.784**	0.689*	0.908**	0.959**	0.894**	1								
*WUE*	−0.848**	−0.795**	−0.850**	–0.452	−0.654*	−0.713**	−0.694*	1							
Chl a	0.881**	0.854**	0.878**	0.621*	0.751**	0.782**	0.815**	−0.821**	1						
Chl b	0.774**	0.838**	0.754**	0.781**	0.838**	0.888**	0.816**	−0.704*	0.730**	1					
Car	0.229	0.325	0.207	0.594*	0.559	0.449	0.583*	–0.22	0.477	0.335	1				
Fv/Fm	0.666*	0.751**	0.642*	0.765**	0.765**	0.777**	0.768**	–0.481	0.558	0.682*	0.49	1			
ΦPSII	0.759**	0.868**	0.729**	0.936**	0.960**	0.919**	0.923**	−0.626*	0.815**	0.834**	0.661*	0.773**	1		
NPQ	−0.803**	−0.871**	−0.781**	−0.781**	−0.869**	−0.909**	−0.885**	0.767**	−0.818**	−0.839**	–0.525	−0.820**	−0.890**	1	
q_P_	0.086	0.21	0.06	0.738**	0.646*	0.442	0.624*	–0.098	0.27	0.408	0.673*	0.331	0.590*	–0.395	1

*Represented significant correlation (*P* < 0.05). **Represented extremely significant correlation (*P* < 0.01).

**TABLE 3:** Correlation analysis between plant growth and photosynthetic parameters of alkali stress under different water potentials.

Correlations	FW	DW	WC	*P*n	*G*s	*C*i	*T*r	*WUE*	Chl a	Chl b	Car	Fv/Fm	ΦPSII	NPQ	q_P_
FW	1														
DW	0.941**	1													
WC	0.998**	0.920**	1												
*P*n	0.792**	0.916**	0.763**	1											
*G*s	0.883**	0.952**	0.861**	0.982**	1										
*C*i	0.931**	0.977**	0.913**	0.932**	0.969**	1									
*T*r	0.900**	0.955**	0.881**	0.958**	0.987**	0.980**	1								
*WUE*	−0.822**	−0.620*	−0.846**	–0.376	–0.54	−0.622*	–0.571	1							
Chl a	0.934**	0.920**	0.926**	0.844**	0.905**	0.955**	0.941**	−0.654*	1						
Chl b	0.945**	0.957**	0.932**	0.872**	0.928**	0.924**	0.921**	−0.665*	0.902**	1					
Car	0.131	0.311	0.101	0.413	0.344	0.391	0.422	0.162	0.364	0.169	1				
Fv/Fm	0.901**	0.938**	0.885**	0.877**	0.907**	0.919**	0.925**	–0.554	0.917**	0.926**	0.38	1			
ΦPSII	0.951**	0.969**	0.937**	0.863**	0.915**	0.964**	0.943**	−.640*	0.973**	0.944**	0.355	0.943**	1		
NPQ	–0.41	–0.352	–0.414	–0.333	–0.368	–0.409	–0.37	0.331	–0.416	–0.432	0.045	–0.306	–0.351	1	
q_P_	0.377	0.594*	0.337	0.836**	0.747**	0.637*	0.711**	0.057	0.501	0.524	0.555	0.549	0.518	–0.302	1

*Represented significant correlation (*P* < 0.05). **Represented extremely significant correlation (*P* < 0.01).

**TABLE 4:** Correlation analysis between plant growth and photosynthetic parameters of drought stress under different water potentials.

Correlations	FW	DW	WC	*P*n	*G*s	*C*i	*T*r	*WUE*	Chl a	Chl b	Car	Fv/Fm	ΦPSII	NPQ	q_P_
FW	1														
DW	0.920**	1													
WC	0.998**	0.894**	1												
*P*n	0.656*	0.861**	0.614*	1											
*G*s	0.696*	0.881**	0.657*	0.988**	1										
*C*i	0.744**	0.830**	0.719**	0.835**	0.851**	1									
*T*r	0.604*	0.800**	0.564	0.981**	0.979**	0.844**	1								
*WUE*	−0.767**	−0.759**	−0.757**	–0.575	−0.684*	−0.657*	–0.575	1							
Chl a	0.846**	0.816**	0.838**	0.796**	0.814**	0.808**	0.813**	−0.634*	1						
Chl b	0.786**	0.951**	0.749**	0.920**	0.915**	0.853**	0.880**	−0.624*	0.768**	1					
Car	0.475	0.644*	0.441	0.850**	0.853**	0.683*	0.890**	–0.52	0.717**	0.711**	1				
Fv/Fm	0.826**	0.884**	0.805**	0.766**	0.775**	0.868**	0.738**	−0.677*	0.805**	0.866**	0.581*	1			
ΦPSII	0.806**	0.872**	0.784**	0.887**	0.881**	0.819**	.0864**	−0.608*	0.931**	0.855**	0.720**	0.894**	1		
NPQ	−0.726**	−0.822**	−0.700*	−0.798**	−0.808**	−0.826**	−0.803**	0.640*	−0.777**	−0.853**	−0.768**	−0.878**	−0.846**	1	
q_P_	–0.02	0.242	–0.062	0.680*	0.625*	0.361	0.726**	–0.011	0.387	0.411	0.715**	0.225	0.474	0.231	1

*Represented significant correlation (*P* < 0.05). **Represented extremely significant correlation (*P* < 0.01).

## Discussion

Early seedling stage plays a vital role in the growth and development of plants, which is greatly sensitive to various environmental abiotic stresses, such as salt, alkali, and drought (Li et al., 2005). In the present study, the fresh and dry weights of *H. jubatum* seedlings under alkali stress were significantly lower than those of salt and drought stresses with the decrease of water potential (Figures 1A,B), indicating that alkali stress had the greatest adverse impacts on the plant morphological characteristics and biomass due to its high pH.

Water is a determining factor for seedling growth of plants. In this study, water potential had significant correlations with many photosynthetic and fluorescent parameters such as *P*n, Fv/Fm, and ΦPSII (Tables 2–4). Plants often suffer from osmotic stress under salt-alkali and drought stresses because of the significant decrease in water potential of the surrounding rhizosphere environment (Verslues et al., 2006). The WC and *WUE* of seedlings are considered as two important indicators to explore the mechanism of physiological response in plants under abiotic stress (Liu and Shi, 2010). Here, the WC decreased and the *WUE* increased in all the stresses, indicating that plant could respond to salt, alkali, and drought stresses by increasing *WUE* and decreasing WC (Figures 1C, 2E). Furthermore, the results also showed that the WC declined most under alkali stress, indicating that alkali stress caused the most serious water loss in the seedlings of *H. jubatum* compared with salt and drought stresses (Figure 1C). This result may be due to that the high pH severely limited water absorption in the roots of seedlings, which broke water balance and then substantially reduced the water transported from roots to the shoots. In addition, the growth parameters of *H. jubatum* seedlings under salt stress were higher than those under alkali and drought stresses. *H. jubatum* has good salt tolerance. It can reduce the absorption of Na^+^ and selectively absorb K^+^ in a high-salt environment to maintain normal physiological processes (Garthwaite et al., 2005). A similar result was also found by Hussain et al. (2020) in *Panicum antidotale.*

Photosynthesis is very sensitive to the environmental stresses and affects seedling growth and stress tolerance of plants (Chaves et al., 2009). In the present study, the photosynthetic gas exchange parameters (*P*n, *G*s, *C*i, and *T*r) were all decreased under salt, alkali, and drought stresses with the decrease of water potential, indicating that photosynthesis of *H. jubatum* seedlings was significantly inhibited by these stresses (Figures 2A–D). Meanwhile, alkali stress also had the lowest photosynthetic capacity at the uniform water potential. This result indicated that the high pH caused by NaHCO_3_ induced much more destructive effects to the photosynthesis of *H. jubatum* seedlings. Similar results have also been reported in the halophyte *Chloris virgata* by Yang et al. (2008). In addition, the results showed that when the *P*n value decreased with the decrease of water potential, the *G*s, *T*r, and *C*i all decreased under salt stress. However, the *C*i showed little change when the *P*n, *G*s, and *T*r decreased at different water potentials under drought stress. Additionally, the *P*n was slightly increased at the highest alkalinity (Figures 2A–D). Farquhar et al. (1980) have earlier reported that the changing trends of *P*n, *G*s, and *C*i consistently indicated that the decreased photosynthetic capacity of plants was mainly due to stomatal factors. Otherwise, the non-stomatal factors play a vital role. Thus, the present research suggested that stomatal factors were the main reason that photosynthesis of *H. jubatum* under salt stress was inhibited. When the roots face high sodium ions environment, it is difficult to absorb water from the soil, and the stomata of the leaf will be closed by signal transduction to reduce the transpiration rate and photosynthetic rate. Moreover, the decreased photosynthetic capacity of this species under alkali stress and drought stress was mainly attributed to non-stomatal factors, such as declined CO_2_ assimilation capacity in mesophyll cells, changed photosynthetic pigments, reduced PSII reaction center activity, and membrane disruption (Albert et al., 2011; Ashraf and Harris, 2013).

Chlorophyll fluorescence can reflect the activity of the reaction center of the photosynthetic system by analyzing plant responses to light absorption and carbon assimilation processes (Basu et al., 2019; Dos Santos et al., 2019). Fv/Fm, the indicator of maximum quantum efficiency of PSII photochemistry, represents maximum efficiency at which light absorbed by PSII is often used for evaluating reduction of Q_A_ and the degree of photoinhibition (Murata et al., 2007). In the present study, the Fv/Fm under salt, alkali, and drought stresses slightly reduced with the decrease of water potential, and alkali stress had the lowest Fv/Fm value (Figure 3A). Thus, these stresses all increased the susceptibility of PSII to photoinhibition and damage of PSII reaction centers, which occurred more severely in alkali stress, especially at the lowest water potential (−0.82 Mpa). ΦPSII is the actual quantum yield of PSII photochemical and is used in photochemistry, which reflects the proportion of light absorbed by chlorophyll related to the PSII (Maxwell and Johnson, 2000). In addition, the photochemical quenching (q_p_) always estimates the redox status of the primary quinone electron acceptor of PSII (Q_A_) and can also indicate the proportion of opened PSII reaction centers (Baker, 2008). In this study, both the ΦPSII and q_p_ in the three stresses were decreased with the decreasing of water potential, and reached the lowest under alkali stress (Figures 3B,C). This indicated that the reduction of photosynthetic activity caused by decreased light absorption for photochemistry and increased PSII negative reaction centers of *H. jubatum* seedlings occurred under the three stresses and seemed more easily affected by alkali stress. The possible reason is that the high pH of NaHCO_3_ much more harmed photosynthesis by damaging oxygen-evolving complex (OEC) on the donor side of PSII, and hindered the electron transfer on the PSII acceptor side (Zhang et al., 2019). Moreover, the ΦPSII and q_p_ values of the three stresses much decreased when the water potential was below −0.47 MPa, especially under alkali stress, indicating that the photosynthetic system was more easily influenced by stresses under lower water potential. Although the Fv/Fm, ΦPSII, and q_p_ values under drought stress were slightly higher than those of salt stress, the differences were not significant, and the *P*n value under salt stress was also significantly higher than that of drought stress, indicating that salt tolerance was stronger than drought tolerance of *H. jubatum* seedlings.

Non-photochemical quenching can afford photoprotection to the photosynthetic apparatus by dissipating excess light energy that cannot be used for photochemistry in the form of heat under stresses (Osmond et al., 2021). In the present study, the NPQ values of both salt and drought stresses increased with the decreasing of water potential and was much higher under salt stress (Figure 3D). This result showed that the seedlings had stronger ability of releasing surplus light energy by heat dissipating to weaken the damage on photosynthetic apparatus under salt stress. However, the NPQ value of alkali stress increased first and then decreased at the lowest water potential, indicating that lower water potential (below −0.47 MPa) caused by high NaHCO_3_ concentration disturbed the photoprotection mechanism of *H. jubatum* seedlings. It is concluded that the xanthophyll cycle depended on the proton gradient across the thylakoid membrane, the xanthophyll Zx and the PsbS protein plays a vital role in photoprotection of NPQ (Jahns and Holzwarth, 2012). Therefore, the reason of NPQ decreased may be that the high pH blocked the xanthophyll photoprotective mechanism, which needs further research.

The photosynthetic pigment content intuitively reflects the photosynthetic performance of plants under stress conditions (Meloni et al., 2003). Chlorophyll generally exists on thylakoid membrane, which has a crucial role for absorption and transmission of light energy in photosynthesis. The previous studies found that plants with less chlorophyll were more susceptible to photoinhibition under various abiotic stresses (Patsikka et al., 2002). Carotenoids play an important role in protecting photosynthetic apparatus against various adverse environmental stresses, such as dissipating excessive light energy, scavenging reactive oxygen species, and enhancing the stability of thylakoid membrane (Strzalka et al., 2003). In the present study, the reduced chlorophyll content values (Chl a, Chl b, and total Chl) were found in the three stresses with the decrease of water potentials, and the most significant groups were under alkali stress among the three stresses (Figure 4). This indicated that the degradation of chlorophyll content in the seedlings of *H. jubatum* occurred when subjected to these stresses. It hindered the photosynthesis process, especially under alkali stress. The reason may be that the high pH of alkali stress makes Fe, Mg, and other essential elements for chlorophyll synthesis reduced effectiveness (Yang et al., 2007). Moreover, it was noted that the most degradation of Chl a content occurred and the Chl a content value of salt stress was lower than drought stress at the lowest water potential (−0.82 MPa). This indicated that this water potential severely disturbed photosynthesis by degrading chlorophyll, and the high concentration of sodium ions under salt stress had more negative effects on the Chl a content than water deficit of drought stress. In addition, the Car content of the three stresses had little increase with the decrease of water potentials, indicating that these stresses all reduced the ability of *H. jubatum* seedlings to dissipate excess light, resulting in a decrease of photosynthetic efficiency. Meanwhile, alkali stress also had the highest value in comparison to other stresses at the uniform water potential, indicating that the high pH of NaHCO_3_ caused more decline to the photochemical assimilation.

## Conclusion

In summary, the result clearly analyzed the comparative effects of salt, drought, and alkali stresses on the growth, photosynthetic, and chlorophyll fluorescence performances in the seedlings of *H. jubatum* based on the same water potential. According to the photosynthetic gas exchange parameters, the decreased photosynthetic rate of *H. jubatum* under salt stress was mainly dominated by stomatal factors, but the non-stomatal factors played a major role under drought and alkali stresses. The chlorophyll content reduced under the three stresses reflected that the absorption and transmission of light energy in photosynthesis was degraded, among which was the most significant under alkali stress. Additionally, the decreased Fv/Fm, ΦPSII, and q_P_ under these stresses indicated that salt, alkali, and drought stresses all increased susceptibility of PSII to photoinhibition, reduced the photosynthetic activity by the declined absorption of light for photochemistry, and increased PSII active reaction centers closed. Moreover, the NPQ was different from the salt and drought stresses under alkali stress, showing that the high pH caused by the NaHCO_3_ disturbed the photoprotection mechanism depending on the xanthophyll cycle. Overall, the *H. jubatum* had stronger tolerance of salt than that of drought and alkali, and the negative effects of alkali stress on growth and photosynthetic performance of this species were most serious.

## Data Availability Statement

The raw data supporting the conclusions of this article will be made available by the authors, without undue reservation.

## Author Contributions

CS wrote the manuscript and did the experiment. FY analyzed the data. ZL and XD did a part of the experiment. YL revised the grammar mistakes. JW and JL conceived and directed the manuscript. All authors contributed to manuscript revision and gave final approval for publication.

## Conflict of Interest

The authors declare that the research was conducted in the absence of any commercial or financial relationships that could be construed as a potential conflict of interest.

## Publisher’s Note

All claims expressed in this article are solely those of the authors and do not necessarily represent those of their affiliated organizations, or those of the publisher, the editors and the reviewers. Any product that may be evaluated in this article, or claim that may be made by its manufacturer, is not guaranteed or endorsed by the publisher.

## Abbreviations

CK: control group
S: salt stress
A: alkali stress
D: drought stress
FW: fresh weights
DW: dry weights
WC: water content
*P*n: net photosynthetic rate
*C*i: intercellular CO_2_ concentrations
*G*s: stomatal conductance
*T*r: transpiration rate
*WUE*: water use efficiency
Chl a: chlorophyll a
Chl b: chlorophyll b
Car: carotenoid
total Chl: total chlorophyll
Φ PSII: the actual quantum yield of photochemical energy conversion in PSII
Fv/Fm: apparent quantum yield of PSII
NPQ: non-photochemical quenching coefficient
q_p_: photochemical quenching coefficient.
